# BlsA integrates light and temperature signals into iron metabolism through Fur in the human pathogen *Acinetobacter baumannii*

**DOI:** 10.1038/s41598-018-26127-8

**Published:** 2018-05-16

**Authors:** Marisel R. Tuttobene, Pamela Cribb, María Alejandra Mussi

**Affiliations:** 1 Centro de Estudios Fotosintéticos y Bioquímicos (CEFOBI- CONICET), 2000 Rosario, Argentina; 2 Instituto de Biología Molecular y Celular de Rosario (IBR-CONICET), 2000 Rosario, Argentina

## Abstract

Light modulates global features of the important human pathogen *Acinetobacter baumannii* lifestyle including metabolism, tolerance to antibiotics and virulence, most of which depend on the short BLUF-type photoreceptor BlsA. In this work, we show that the ability to circumvent iron deficiency is also modulated by light at moderate temperatures, and disclose the mechanism of signal transduction by showing that BlsA antagonizes the functioning of the ferric uptake regulator (Fur) in a temperature-dependent manner. In fact, we show that BlsA interacts with Fur in the dark at 23 °C, while the interaction is significantly weakened under blue light. Moreover, under iron deprived conditions, expression of Fur-regulated Acinetobactin siderophore genes is only induced in the dark in a BlsA-dependent manner. Finally, growth under iron deficiency is supported in the dark rather than under blue light at moderate temperatures through BlsA. The data is consistent with a model in which BlsA might sequester the repressor from the corresponding operator-promoters, allowing Acinetobactin gene expression. The photoregulation of iron metabolism is lost at higher temperatures such as 30 °C, consistent with fading of the BlsA-Fur interaction at this condition. Overall, we provide new understanding on the functioning of the widespread Fur regulator as well as short-BLUFs.

## Introduction

BLUF (Blue Light Using Flavin) domains are light-triggered switches that control enzyme activity or gene expression in response to blue light^1^. These photosensors bind riboflavin (RB) derivatives as chromophores, namely FMN (Flavin Mononucleotide) or FAD (Flavin Adenine Dinucleotide)^2^. Many BLUF proteins, including AppA and PAC, carry an extra domain adjacent to the BLUF domain, with enzymatic activity or other properties^1^. However, the majority of bacterial BLUF proteins, do not possess an effector domain covalently linked^2^, and consist solely of the BLUF core and a C-terminal extension^1^. The BLUF domain transmits the light-induced signal to downstream protein modules via intermolecular or intramolecular interactions^3^. Despite being widely distributed in bacterial genomes, signal transduction involving BLUF photoreceptors has been envisaged only in a few cases.

We have previously recognized that *Acinetobacter baumannii* perceives and responds to light modulating global features of its physiology through the only “traditional” photoreceptor encoded in its genome, a short BLUF protein designated BlsA^4–6^. *A. baumannii* is in fact a threatening human pathogen considered to be the paradigm of multidrug resistance (MDR), and recognized recently as one of the pathogens requiring urgent attention by the world health organization (WHO). A key component in its success as a pathogen is its outstanding capability to acquire resistance as well as its peculiar ability to reside and persist in the hospital environment, factors that contribute to colonization, infection, and dissemination of the MDR clones^7,8^.

Cellular processes modulated by light in *A. baumannii* are wide and diverse. In fact, we have previously shown that in *A. baumannii* light modulates motility, biofilm formation, and virulence against *Candida albicans*^4^. We further showed that light regulates metabolic pathways such as the phenylacetic acid degradation pathway and trehalose biosynthesis, susceptibility and tolerance to some antibiotics, antioxidant enzyme levels such as catalase, likely contributing to bacterial persistence in adverse environments^6,9^. Moreover, the expression of whole pathways and gene clusters, such as genes involved in lipid metabolism, the complete type VI secretion system, as well as efflux pumps related to antibiotic resistance, were differentially induced by light^6^. In *A. baumannii*, light modulation occurs at moderate temperatures such as 23 °C but not at higher such as 37 °C, in contrast to other *Acinetobacter* species^4,5,10^. It is therefore clear that light could have a direct effect on persistence of the bacteria in the environment. On the contrary, light may not play a role in systemic infections in humans but rather might be relevant in the pathogenesis of surface-exposed wound infections, considering the exposure of the bacteria to light and the relatively lower temperatures of skin wounds^4^. Besides BlsA, no other clues on the mechanism or other components of the light signal transduction are known for this pathogen.

In this work, we provide for the first time evidence on the mechanism of light signal transduction mediated by BlsA in *A. baumannii* by showing that this photoreceptor directly interacts with the ferric uptake regulator, Fur, antagonizing its functioning and enabling expression of the Acinetobactin siderophore gene cluster, as well as growth under iron deprived conditions.

Iron is involved in diverse functions in bacterial cells^11^. However, an excess of Fe(II) is toxic for the cell because of the formation of highly reactive radicals via the Fenton reaction, and therefore, the intracellular iron concentration has to be tightly regulated^11^. In mammals, iron is virtually unavailable to invading bacteria, being mainly incorporated into iron transport and storage proteins. One of the strategies developed by bacterial pathogens to respond to iron deprivation imposed by the human host is the production of high-affinity uptake systems known as siderophores^12^. Siderophores, such as *A. baumannii* Acinetobactin, are low molecular weight compounds which solubilize external Fe^+3^, and whose production is strongly repressed by the presence of iron. In Gram-negative bacteria, iron-regulated gene expression is generally under the control of Fur^11,13,14^. The binding of Fe(II) induces a conformational change that allows Fur to behave as a transcriptional repressor by blocking access of RNA polymerase to the DNA.

Most of the genes involved in Acinetobactin biosynthesis and utilization are organized in a genomic cluster^15^ identified by Fur titration assay (FURTRA) in *A. baumannii* ATCC 19606, i.e. by the ability of the Fur repressor to bind to the corresponding Fur boxes in the promoter-operator regions, followed by cloning and sequencing of the sequestered DNA fragment^15^. Subsequent studies determined that the cluster includes 18 genes expressed in seven transcriptional units from five Fur-related promoters^15^. Some of these genes are involved in Acinetobactin biosynthesis and were designated *bas* (A. *baumannii* Acinetobactin biosynthesis); others *bau*, which stand for *A. baumannii* Acinetobactin utilization; and finally the *barAB* genes, which are involved in Acinetobactin release out of the cell (*A. baumannii* Acinetobactin release)^15^. Functional analysis of the *bauA* and *basD* mutants proved that the Acinetobactin-mediated system is the only siderophore-mediated high-affinity iron acquisition system produced by the type strain 19606 under iron deprived conditions^12,16,17^.

Overall, in this work we provide new understanding of iron homeostasis, showing that light modulates iron acquisition and revealing a new role for Fur in transduction of the light signal mediated by BlsA.

## Results

### BlsA interacts with Fur in the dark at moderate temperatures in *A. baumannii*

To find BlsA´s partners that mediate signal transduction between light perception and transcriptional regulation by protein-protein interactions, we performed a candidate approach. To do this, we first surveyed the literature and databases for confirmed or proposed transcriptional regulators of the pathways that we have shown before to be modulated by light through BlsA^6^. One of these candidates was the Fur regulator, as in other microorganisms it was shown to regulate catalase^18^; and also as enzymes involved in the phenylacetic acid degradation as well as benzoate degradation pathways were shown to be modulated by iron levels in *A. baumannii*^14^.

**Figure 1:**
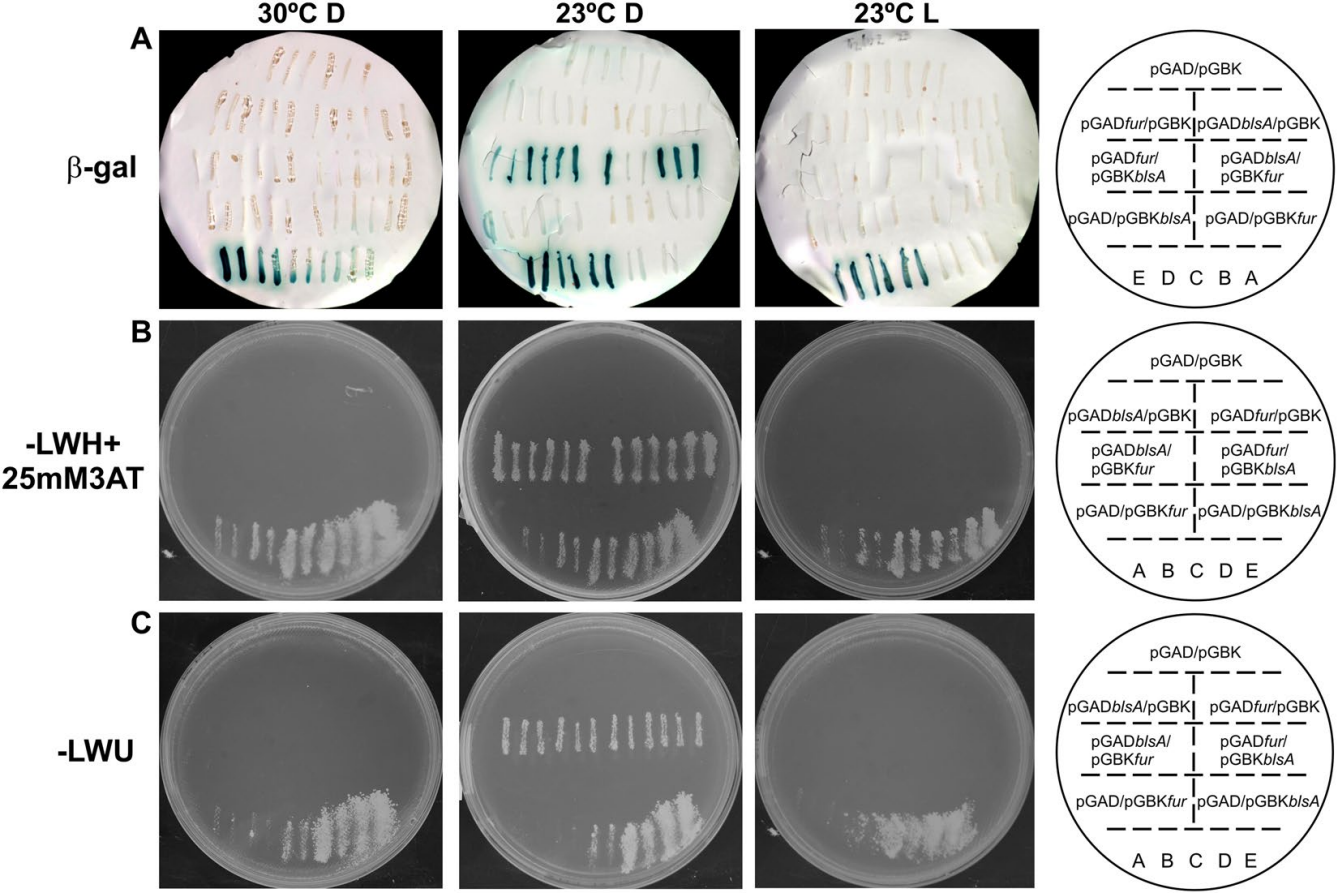
BlsA interacts with Fur at moderate temperatures in the dark, but not under blue light or at higher temperatures. Y2H assays of BlsA/Fur interaction at the different conditions, namely 23 °C in the dark (D), 23 °C under blue light (L) or 30 °C in the dark. Each plate contains six clones of MaV203/pGAD-*blsA* or MaV203/pGAD-*fur* transformed with plasmids pGBK-*fur* or pGBK-*blsA*, respectively, as well as plasmid pGBK as negative control. In addition, we included at least six clones of MaV203/pGBKT-*blsA* or MaV203/pGBKT-*fur* transformed with plasmid pGAD as negative control. Finally, two clones of each interaction-control strain A–E were also included. Pictures show development of blue color (panel A) or yeast growth in Synthetic Complete medium (SC) without leucine, tryptophan and histidine (SC-LWH) supplemented with 25 mM 3-amino-1, 2, 4-triazole (3AT) (panel B), and SC without leucine, tryptophan and uracil (SC-LWU) (panel C). For β-galactosidase expression analysis (X-Gal), yeasts were transferred to a nitrocellulose filter, permeabilized and subjected to the X-Gal assay. The scheme on the right side represent the order of yeast streaks on each plate. The assays were performed following procedures described in^19^. Experiments were performed in triplicates and representative results are shown.

To investigate whether BlsA interacts with Fur, we performed yeast two hybrid experiments (Y2H) using a system adapted from the ProQuest™ Two-Hybrid System (http://www.invitrogen.com/content/sfs/manuals/10835031.pdf). The yeast strain employed, Mav 203, contains three different reporter genes, namely *lacZ* and two auxotrophic markers such as HIS3 or URA3, with independent promoters to rapidly weed out false positives. Whether an interaction occurs, the system would result in the development of blue color or gain in the ability of the yeast to grow in the absence of histidine or uracil. Based in our previous experience^19^, we used Gateway-system adapted Y2H vectors pGAD-T7Gw and pGBK-T7Gw to express each gene under study, namely *blsA* and the single *fur* homolog produced by *A. baumannii*^13,20,21^, as fusions to the GAL4 DNA binding domain (DB) and activation domain (AD), respectively. Self-activation controls (pGAD-T7Gw and pGBK-T7Gw empty vectors) and different force interaction controls (A–E) were also included in each plate, allowing for a better comparison between the reporter genes’ expression levels. Figure 1 summarizes the Y2H assay results for the different reporter genes at different conditions, which in all cases provided congruent results. At 23 °C in the dark (Fig. 1), the three reporters showed positive indications for the interaction between BlsA and Fur, evidenced by the development of blue color, as well as the ability to grow in plates lacking histidine or uracil. As expected, the reporter gene expression was observed for both the pGAD*blsA*/pGBK*fur* and pGAD*fur*/pGBK*blsA* clones. It is noteworthy that growth was observed even on SC-Ura plates, since only strong protein:protein interactions can induce the least sensitive *URA3* reporter gene sufficiently to allow growth, therefore indicating that the interaction between BlsA and Fur is strong under these conditions. The possible self-activation of each protein fused to DB was tested in strains expressing the GAL4 AD alone and the corresponding fusion to DB (pGAD-T7/pGBK*blsA* or pGBK*fur*). In addition, AD fusions (pGBK-T7/pGAD*blsA* or pGAD*fur*) were also tested and, as expected, none of those were capable of activating reporters’ expression by themselves. These results allow us to confirm that BlsA interacts with Fur specifically at 23 °C and in the dark. On the contrary, at 23 °C under blue light, Y2H assays showed null or negligible Fur-BlsA interaction for the three reporters (Fig. 1). Overall, our results show that BlsA interacts with Fur in a light dependent manner at moderate temperatures.

Furthermore, we also assayed self interactions of BlsA or Fur. In the case of BlsA, interaction BlsA-BlsA was observed for the three reporters at all conditions assayed, i.e. 23 °C under blue light or in the dark and 30 °C in the dark (see Fig. S1), indicating an important tendency of BlsA to form oligomers. On the contrary, Fur-Fur interactions were found to depend on the presence of iron. Indeed, Fur-Fur interaction allowed the reporter genes’ expression in the presence of 0.75 mM FeCl_3_ in the growth medium under all conditions tested, however, the interaction appears to be impaired in the absence of added iron (see Fig. S2).

### Expression of the Acinetobactin gene cluster is modulated by light and depends on BlsA at moderate temperatures in *A. baumannii*

**Figure 2:**
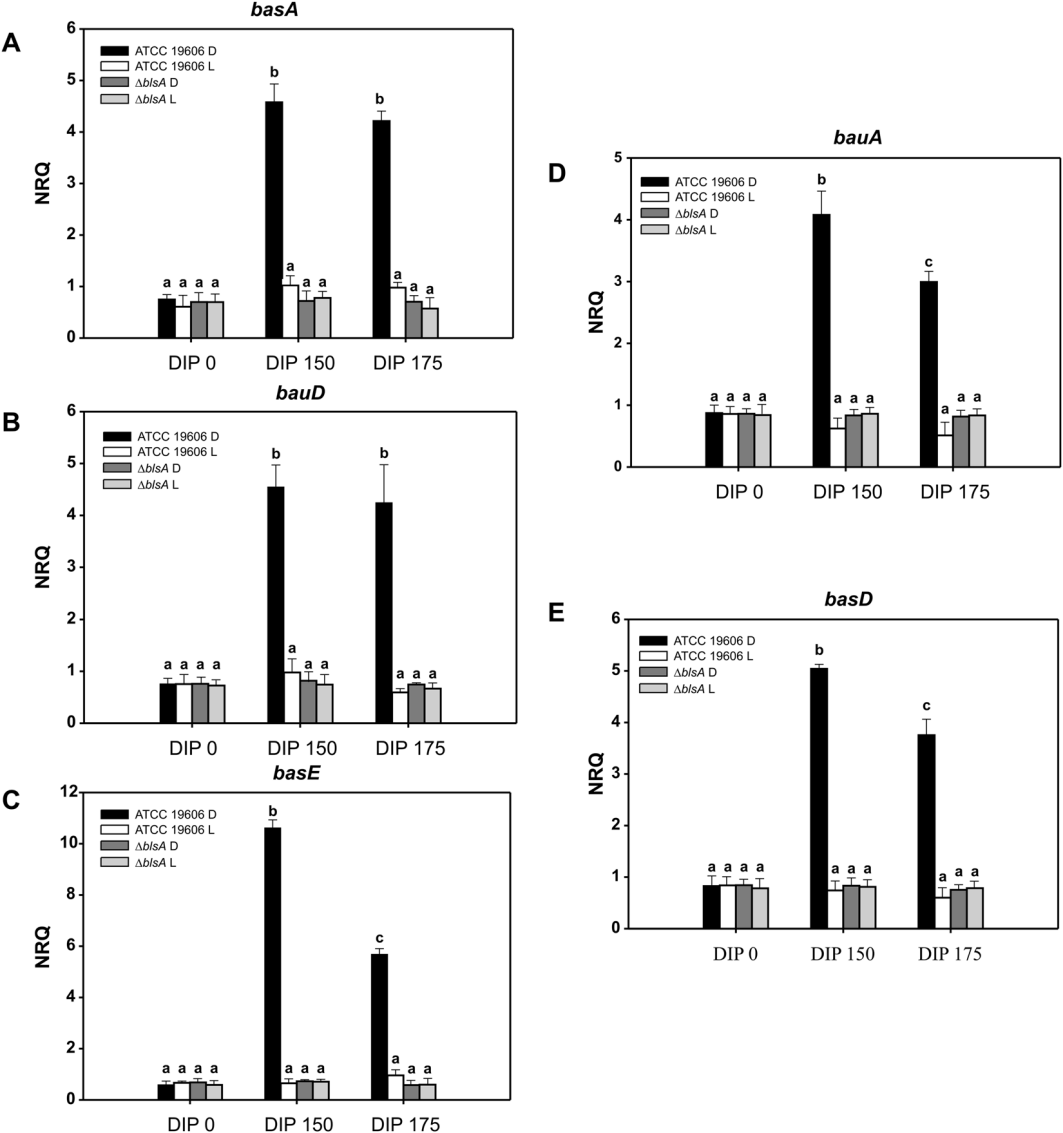
Acinetobactin genes are induced in the dark at moderate temperatures under iron deprived conditions. Estimation by RT-qPCR of the expression levels of representative genes components of the Acinetobactin siderophore cluster, namely *basA*, *bauD*, *basE*, *bauA* and *basD*, in ATCC 19606 wild-type and *blsA* genetic backgrounds at 23 °C under blue light (L) or in the dark (D). The data shown are mean ± SD of normalized relative quantities (NRQ) obtained from transcript levels of representative Acinetobactin cluster genes such as *basA*, *bauD*, *basE*, *bauA* and *basD*, in samples grown in LB at the indicated DIP concentrations under blue light or in the dark at 23 °C, measured in at least three biological replicates. Different letters indicate significant differences as determined by ANOVA followed by Tukey’s multiple comparison test (p < 0.05).

Since our data indicate that BlsA interacts with Fur at 23 °C in the dark, we next analyzed the expression of Fur-regulated Acinetobactin cluster genes to follow Fur activity under different conditions. For this purpose, we analyzed the expression of *basA*, *bauD, basE*, *bauA* and *basD* by qRT-PCR at different light and iron-availability conditions in strain ATCC 19606. Our results show that their expression levels were basal both under blue light or in the dark at 23 °C in LB, a condition in which iron is not limiting (DIP = 0 µM). However, under iron deprived conditions (DIP 150 or DIP 175 µM), the expression of the studied genes was induced only in the dark (Fig. 2), while under blue light the expression levels were similar to those observed for the non iron-limiting condition (Fig. 2). Therefore, the Acinetobactin gene expression was unresponsive to iron limitation under blue light at 23 °C, maintaining basal levels similar to those observed in the iron proficient condition, i.e. as if the repressor was bound to the promoter region. On the contrary, in the dark, the system responded to iron deprivation inducing the corresponding expression of siderophore genes at moderate temperatures.

In the *blsA* mutant, the expression of these genes was similar under blue light or in the dark at 23 °C under iron limiting conditions, and comparable to those observed for the non iron-deprived condition (LB DIP = 0 µM), i.e., without induction in the dark. These results show that modulation by light of the expression of genes of the Acinetobactin cluster is dependent on the presence of the BlsA photoreceptor. In other words, the Acinetobactin genes are expressed at a basal level at 23 °C (repressed) even under iron deprived conditions (Fig. 2), had it not been for the presence of BlsA that rescues this inhibition in the dark. The overall data so far is consistent with a model in which BlsA interacts with Fur in the dark at 23 °C, antagonizing the repressor functioning and resulting in induction of the expression of the Fur-regulated, Acinetobactin gene cluster.

### Growth under iron limitation is modulated by light and depends on BlsA at moderate temperatures in *A. baumannii*

**Figure 3:**
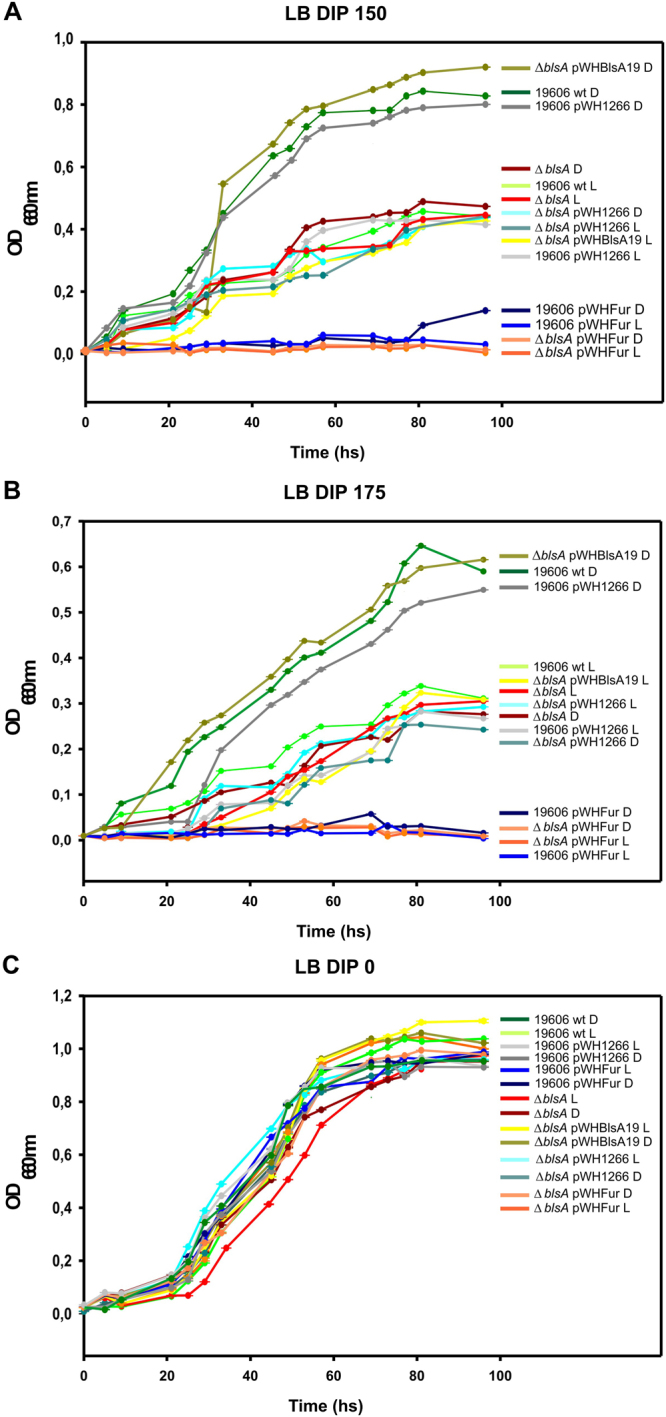
Light modulates growth at moderate temperatures under iron deprived conditions in *A. baumannii* ATCC 19606. (**A**) Growth curves of *A. baumannii* ATCC 19606 wild-type and derivative strains used in this study in LB medium supplemented with DIP 150 or 175 µM incubated stagnantly at 23 °C under blue light (L) or in the dark (D). The experiment was repeated at least three times, including in each repetition three replicates for each strain at each condition. Representative results are shown.

The evidence obtained above indicating that Fur interacts with BlsA at 23 °C in the dark with the concomitant induction of expression of Acinetobactin genes only in this condition, suggested that light could be modulating iron metabolism. Therefore, we next studied whether the ability to acquire iron was modulated by light by performing growth curves in LB under iron deprived conditions in the dark or under blue light at 23 °C. Our results show that, under iron deprived conditions, strain ATCC 19606 displays a much poorer capability to grow under blue light rather than in the dark at 23 °C (Fig. 3A,B). The *blsA* mutant, both under blue light and in the dark, showed a similar pattern as the wild type under blue light, as did the mutant harboring the empty pWH1266 (Fig. 3A,B). On the contrary, the mutant harboring plasmid pWHBlsA19, which expresses a wild type copy of *blsA* from its own promoter, complemented the *blsA* mutation as the ability to grow under iron deprived conditions better in the dark than under blue light was restored (Fig. 3A,B). Reducing the levels of a particular protein in the cell by generating isogenic mutants or increasing its levels by overexpression are strategies frequently used to study the function a particular gene plays in the bacterial cell. Given that *fur* could not be knocked out neither by us nor by others despite many attempts^22,23^, therefore suggesting that *fur* is an essential gene in *A. baumannii*, we constructed a plasmid expressing its coding sequence from its own promoter and assayed the effect on growth under iron deprived conditions. As shown in Fig. 3A,B, the presence of higher levels of Fur resulted in complete abolition of the bacterial capacity to grow under low iron, regardless of the presence of light or in the dark, or BlsA content. It should be noted that there is no effect of light on the growth of cells under iron replete conditions, as the growth curves were similar under blue light or in the dark for the different strains in LB (Fig. 3C). Overall, the data is consistent with light modulation of iron acquisition in ATCC 19606, and the response depends on the BlsA photoreceptor. To reinforce this notion, similar experiments performed using tryptone media instead of LB and also strain ATCC 17978 in addition to ATCC 19606, showed modulation by light as well as dependence of the light response on BlsA (see Fig. S3).

### BlsA-Fur interaction is significantly reduced at higher temperatures

In *A. baumannii*, light modulation occurs at moderate temperatures such as 23 °C but does not take place at higher temperatures such as 30 or 37 °C (^4–6^ and unpublished results). BlsA-Fur interaction was verified at 23 °C in the dark, and the question therefore arises regarding whether the interaction is maintained at higher temperatures. Therefore, we next assayed BlsA-Fur interactions by Y2H but incubating the plates at 30 °C in the dark. A control at 23 °C in the dark was also included for each repetition. Representative results are shown in Fig. 1. Y2H assays show a negligible Fur-BlsA interaction at higher temperature conditions (Fig. 1).

Our results therefore show that BlsA-Fur interactions occur at certain physiological conditions, which are coincident with conditions of photoregulation observed so far, i.e. 23 °C but not higher temperatures such as 30 °C.

### Modulation of iron metabolism by light does not occur at higher temperatures

**Figure 4:**
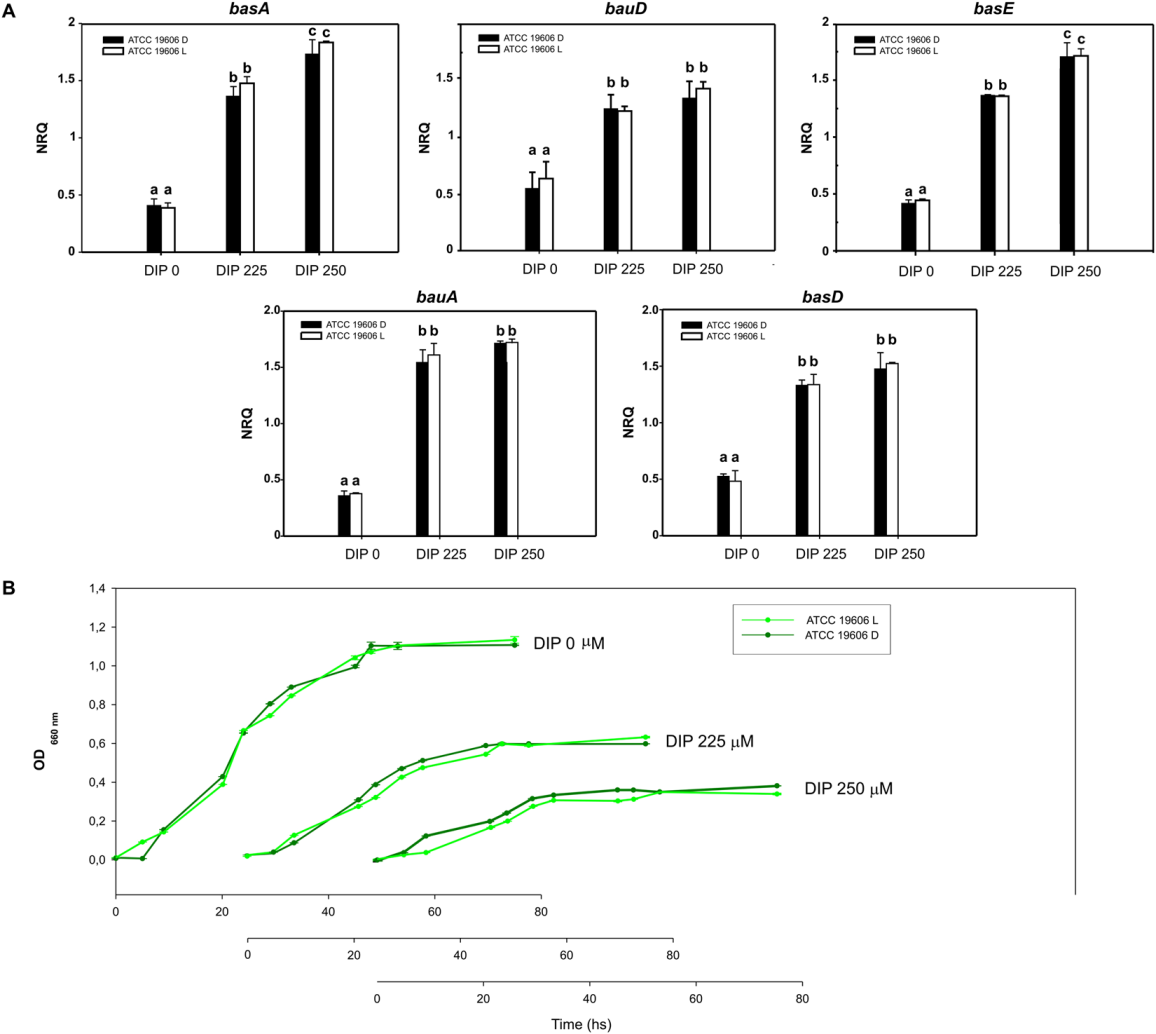
Modulation of iron metabolism by light does not occur at higher temperatures. (**A**) Estimation by RT-qPCR of the expression levels of representative genes components of the Acinetobactin siderophore cluster, namely *basA*, *bauD*, *basE*, *bauA* and *basD*, in ATCC 19606 at 30 °C under blue light (L) or in the dark (D). The data shown are mean ± SD of normalized relative quantities (NRQ) obtained from transcript levels of representative Acinetobactin cluster genes such as *basA*, *bauD*, *basE*, *bauA* and *basD*, in samples grown in LB at the indicated DIP concentrations under blue light or in the dark at 30 °C, measured in at least three biological replicates. Different letters indicate significant differences as determined by ANOVA followed by Tukey’s multiple comparison test (p < 0.05). (**B**) Growth curves of *A. baumannii* ATCC 19606 wild-type LB medium supplemented with DIP 0, 150, 225 or 250 µM incubated stagnantly under blue light or in the dark at 30 °C. Growth was measured by determining the optical density at 660 nm. The curves are inserted into the same graphic sharing Y axis, while the x axes we added for each curve at the bottom.

Our results indicating that BlsA and Fur show null or negligible interaction at 30 °C prompted us to study Acinetobactin gene expression and growth under iron deprived conditions at this temperature. As expected, the siderophore-related genes, namely *basA*, *bauD*, *basE*, *bauA* and *basD*, were more expressed under iron deprived conditions (DIP 225 and 250 µM) relative to the iron sufficient condition (DIP 0 µM). However, in accordance with the observed lack of interaction between BlsA and Fur at 30 °C, expression of the Acinetobactin genes showed no differential modulation by light (Fig. 4A). In addition, growth curves performed in LB with increasing concentrations of DIP showed no significant difference between light and dark (Fig. 4B). Overall, these results indicate that iron acquisition is not subjected to light modulation at 30 °C, and are congruent with the previous notion that photoregulation occurs only at moderate temperatures in *A. baumannii*^4,5,10^. It should be noted that at this higher temperature, higher DIP concentrations had to be used to obtain an effect of iron deprivation on growth relative to 23 °C. A similar situation occurred with the expression of the Acinetobactin cluster genes, whose induction occurred at higher DIP concentrations at this temperature, DIP = 225 and 250 µM, rather than at DIP 150 and 175 µM as occurred at 23 °C (not shown). This is not surprising since it is well known that susceptibility to antibacterial agents depends on the culture conditions such as media composition, temperature, etc.

### Temperature modulates expression of Acinetobactin genes under iron deprivation

**Figure 5:**
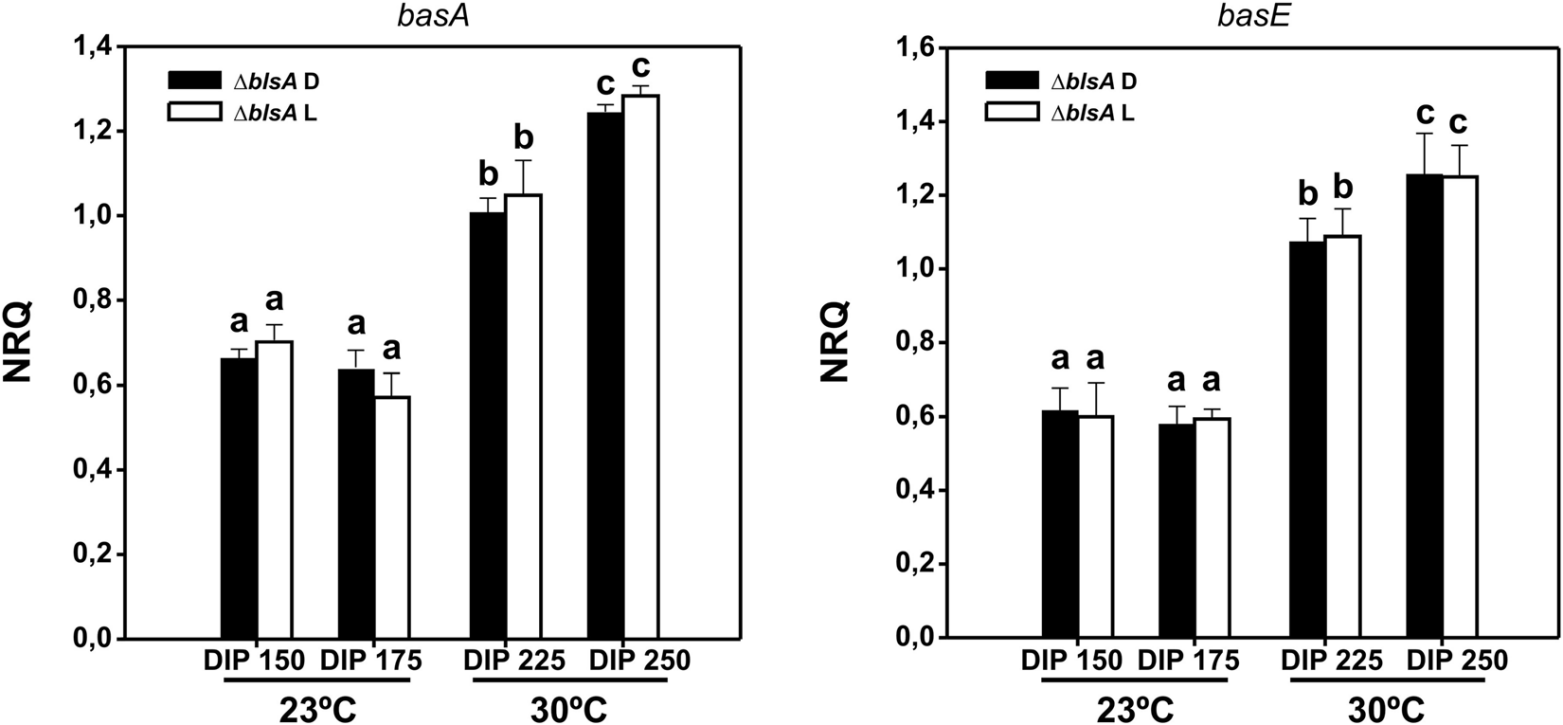
Temperature modulates expression of Acinetobactin genes under iron deprivation. Estimation by RT-qPCR of the expression levels of representative genes components of the Acinetobactin siderophore cluster, namely *basA* and *basE*, in ATCC 19606 *blsA* genetic background at 23 °C and 30 °C under blue light (L) or in the dark (D) under iron deprived conditions. The data shown are mean ± SD of normalized relative quantities (NRQ) obtained from transcript levels of *basA* and *basE* in samples grown in LB at the indicated DIP concentrations under blue light or in the dark at 23 °C and 30 °C, measured in at least three biological replicates. Different letters indicate significant differences as determined by ANOVA followed by Tukey’s multiple comparison test (p < 0.05).

We further compared the levels of expression of representative Acinetobactin genes, such as *basA* and *basE*, in *A. baumannii* ATCC 19606 *blsA* background at 23 and 30 °C under iron deprived conditions. We evaluated light vs. dark but, as expected, no differences were observed between the two conditions, as photoregulation is lost given to the absence of BlsA (Fig. 5). As also shown in the figure, at 30 °C *basE* and *basA* expression levels were 1.5 to 2 fold higher than at 23 °C under iron deprived conditions. Overall, the data suggests that temperature modulates Fur binding to Acinetobactin promoters. In fact, the results are compatible with more Fur molecules binding to promoters (or with higher affinity) at 23 °C than at 30 °C under iron deficient conditions. Other possibilities are also plausible such as the differential presence of a yet unknown factor at 23 °C contributing to Fur binding to promoters. It should be noted that, as was explained in the above item, the minimal inhibitory concentrations for DIP vary with culturing temperature, and different DIP conditions were required to produce iron deprivation at 23 °C (DIP 150 and 157 µM) and at 30 °C (DIP 225 and 250 µM).

## Discussion

**Figure 6:**
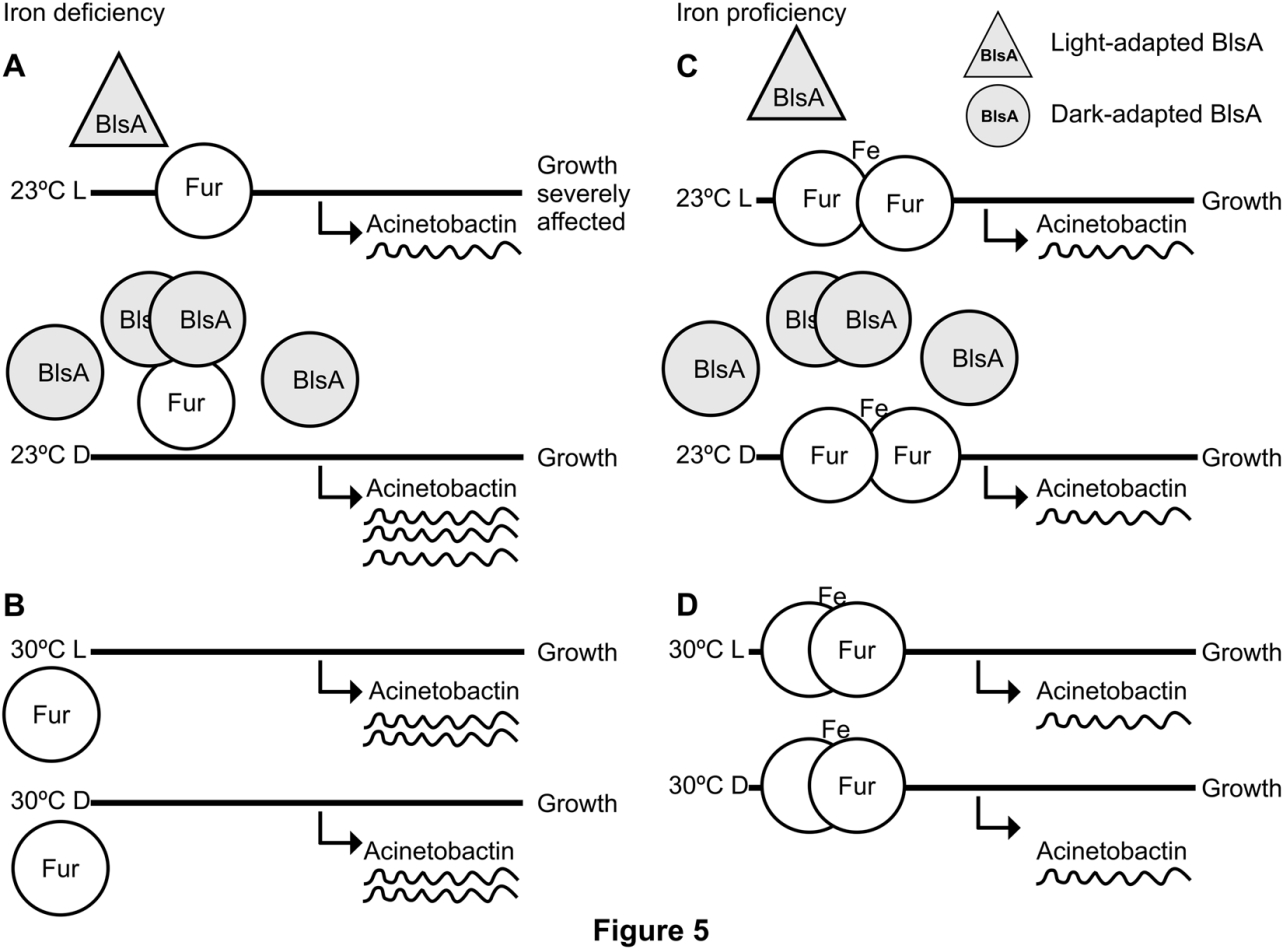
Working model depicting Fur and BlsA behavior in iron homeostasis at different physiological conditions. Under iron deficiency at 23 °C (Fig. 5A), Fur would not form oligomers and might be able to bind operator regions with low affinity. In the dark, BlsA is produced in higher quantities than under blue light, and interacts strongly with Fur antagonizing its activity as repressor. As a result, Acinetobactin gene expression is induced and growth is supported under iron deprived conditions. Under blue light, BlsA is in an excited state and its levels are much more reduced than in the dark, and does not support the interaction with Fur. Therefore, at this condition more Fur molecules are able to bind to Acinetobactin gene promoters and growth is severely affected. Overall, BlsA performs fine tuning of Fur levels available to bind Acinetobactin promoters at 23 °C under iron deprived conditions. Under iron deprivation conditions at 30 °C (Fig. 5B), Fur would not form oligomers and is basically released from Acinetobactin promoters, being the corresponding genes induced and growth is supported. Under iron proficient conditions (Fig. 5C,D), Fur forms oligomers with high affinity (much more than with BlsA), independently of the illumination and temperature condition, favoring its binding to Acinetobactin promoters and expressing the cluster genes at basal levels.

The signal transduction mechanism of BlsA has remained elusive even though it is directly involved in regulation by light of many cellular processes in *A. baumannii*^4,6^. In this work, we disclose a new physiological trait modulated by light in this pathogen as is the ability to circumvent iron deprivation, and provide for the first time insights into the signal transduction mechanism of BlsA by showing that this photoreceptor integrates a light signal into iron metabolism through association with Fur. First, we showed that BlsA interacts with Fur at 23 °C in the dark by Y2H experiments. Then, expression of the genes coding for the siderophore Acinetobactin are modulated by light showing induction only in the dark and dependence on BlsA. Finally, we also showed that the ability to grow under iron deprivation is modulated by light and depends on BlsA, and again, bacterial growth is supported in the dark while under blue light it is severely affected. The overall data is consistent with a model in which BlsA binding to Fur at 23 °C in the dark antagonizes the repressor functioning, likely reducing its binding to Acinetobactin promoters and allowing expression of the corresponding genes at this condition (Fig. 6A). Another important factor to be taken into account is BlsA levels at the different physiological conditions. In fact, BlsA levels are much higher in the dark than under blue light at 23 °C (Fig. S4 and^4,10^), and this could result in a higher ability of BlsA to sequester Fur in the dark rather than under blue light (Fig. 6A). Fur levels available in the cell to bind promoters therefore appear important for the regulation, as is also observed in growth curves (Fig. 3A,B). Therefore, BlsA seems to perform a fine tuning of Fur levels available to bind promoters *in vivo* at 23 °C. Another important aspect is that under blue light BlsA is subjected to an excited state that could confer differential properties regarding its ability to bind Fur, as well as its own oligomerization state. Our data suggest that BlsA forms oligomers both under blue light or in the dark at 23 °C (Fig. S1). However, these complexes could vary in the composition and order level at each condition. At 30 °C, the expression of Acinetobactin genes is unresponsive to light, while induced under iron deprived conditions (Fig. 6B–D). Since the levels of the photoreceptor are much reduced at higher temperatures such as 37 °C^4,10^ or 30 °C (Fig. S4), this could also contribute to the absence of photoregulation observed. Indeed, our results show that *blsA* expression levels are very low in the cells at 30 °C (Fig. S4), and at 37 °C BlsA levels are negligible^10^. Furthermore, our Y2H results show that had BlsA been present at 30 °C, as one could expect in a physiological situation of sudden temperature change, it would not interact with Fur (Fig. 1). For many bacteria, it has been reported that binding of iron (ferrous iron) to the regulatory site of the Fur dimer enables the protein to bind to Fur box, thus repressing gene transcription^11,24^. This could also be the case in *A. baumannii*, as our Y2H results indicate that Fur self interaction is favored in the presence of iron (Fig. S2). This interaction could indeed be much more stronger than the Fur-BlsA interaction in iron proficient conditions, supporting binding of Fur to promoters with high affinity, and resulting therefore in a basal expression of the Acinetobactin genes at this condition (Fig. 6C,D), regardless of the temperature. Whether Fur is able to bind Acinetobactin promoters at 23 °C under blue light even under iron deprived conditions, should be further explored, but seems a plausible possibility given our results showing basal Acinetobactin expression levels and severely affected growth. Indeed, repression of gene expression by apo-Fur has already been reported in *Helicobacter pylori*^25^ despite this apo-repression appears to depend on the presence of an additional α-helix at the N-terminus of the Fur protein, conserved in the proteobacteria clade^26^. Also, regulation of Fur binding by oxidative signals has also been described^25^. In the same context, the possibility that Fur binding to Acinetobactin promoters is modulated by termperature under blue light and low iron conditions (Fig. 5), should also be further studied, and could imply changes of Fur affinity to promoters with temperature

The mechanism of light signal transduction has been characterized for a few BLUF-domain containing proteins^1,27–29^. Some of them have also been reported to interact and antagonize the functioning of transcriptional regulators, as described here for BlsA of *A. baumannii*. For example, AppA from *Rhodobacter spharoides*, which harbors a SCHIC (Sensor Containing Heme Instead of Cobalamin) domain covalently attached to the BLUF domain, interacts with the DNA binding protein PpsR to control photosynthesis^30^. AppA–PpsR complex formation in the dark or under low light antagonized PpsR binding to its target DNA promoters^29–31^. Once AppA is activated by blue light illumination, it undergoes a conformational change and dissociates from PpsR, thereby inhibiting photosynthetic gene transcription. In *E. coli*, the BLUF domain of YcgF is associated with a C-terminal pseudo-EAL domain. YcgF directly binds to the MerR-like repressor YcgE and releases it from its operator DNA in a light-dependent manner, which can ultimately modulate biofilm functions^32^. Therefore, these two BLUF-containing proteins also bind transcriptional repressors and antagonize their functioning. Regarding short BluFs, the most extensively characterized is PixD from *Synechosystis* sp. PCC 6803, which is involved in controlling phototaxis by interacting with the response regulator-like protein PixE in an apparent light intensity-dependent manner^27,33,34^. Under dark conditions PixD forms a large molecular weight complex with PixE^27^, inhibiting PixE’s ability to prevent positive phototaxis. Upon photoexcitation of two PixD molecules in a complex, PixE is released from the complex and positive phototaxis is then inhibited^27,34^. The evidence so far suggests that the PixD–PixE complex could regulate phototaxis through type-IV pili and the motor protein PilB, although it is not clear how this is accomplished^27,35^. These examples illustrate the diversity of mechanisms and components of light signal transduction in bacterial cells, also disclosing the novelty of signal tranduction by BlsA.

Many questions arise from our findings such as why is the ability to grow under iron deficiency photoregulated at moderate temperatures? Why has this pathogen evolved such a sophisticated mechanism that enables it to grow under iron deprivation in the dark at moderate temperatures? Or rather, why is growth under iron deprivation restricted in the presence of light? The answer may reside in the lifestyle the microorganism leads at this condition. One would expect it to live outside the human host at 23 °C, and be exposed to environmental fluctuations such as presence/absence of light, temperature and nutrient composition shifts, etc. In the presence of light, the production of reactive oxidative species can be stimulated by a superoxide-driven Fenton reaction (or iron-catalyzed Haber-Weiss reaction)^36^. Moreover, it has been shown that irradiation with UV-visible light accelerates Fenton reaction by photoreduction of Fe(III) back to Fe(II) (photo-Fenton) *in vitro*^37^. Therefore, restraining iron bioavailability under light conditions could constitute a strategy to avoid oxidative stress and increase antioxidant tolerance which is, indeed, a universal strategy. Perhaps at higher temperatures compensatory mechanisms like antioxidant defenses are more efficient and could explain absence of photoregulation of iron acquisition at this condition. This hypothesis needs further exploration.

Whether BlsA interacts with Fur to modulate other physiological traits such as those previously shown in *A. baumannii* to be regulated by light, or rather, BlsA interacts with different partners to accomplish these functions is currently under study in our laboratory. Indeed, a global role has been described for Fur in various literature reports, indicating that in addition to regulation of iron metabolism, Fur could also modulate many other cellular processes. *Escherichia coli* Fur, in particular, is a global transcriptional regulator that controls the expression of more than 90 genes mainly implicated in iron uptake but also in other fundamental processes, such as the regulation of oxidative stress, acid tolerance, and bacterial virulence determinants^24^. Indeed, as mentioned before, it has been shown that Fur regulates catalase, and the levels of enzymes involved in the phenylacetic acid degradation pathway and benzoate pathway are modulated by iron^14^. The question arises on whether Fur and BlsA also act in concert to regulate these phenotypes.

Overall, the mechanism of Fur functioning appears to be complex and variable across the different bacterial species, and *A. baumannii* appears not to be an exception. In this work, we contribute to iron homeostasis knowledge also at environmental temperatures, which has not been much studied so far.

## Methods

### Bacterial strains, plasmids, and media

The bacterial strains and plasmids used in this work are listed in Table S1. Luria-Bertani (LB) broth (Difco) and agar (Difco), as well as tryptone media (tryptone 1%, NaCl 0.5%, agarose 0.3%) were used to grow bacterial strains. When indicated, the iron quelator 2,2′-dipyridyl (DIP) was added to the media. Broth cultures were incubated either statically or with shaking at 200 rpm at the indicated temperatures.

### Blue light treatments

Blue light treatments were performed as described in our previous studies^4–6,10^. Briefly, cells were incubated at 23 or 30 °C in the dark or under blue light emitted by 9-light-emitting diode (LED) arrays, with an intensity of 6 to 10 µol photons/m^2^/s. Each array was built using 3-LED module strips emitting blue light, with emission peaks centered at 462 nm, determined using a LI-COR LI-1800 spectroradiometer^4^.

### Plasmid construction

#### Y2H Plasmid Construction

*blsA* and *fur* coding sequences were amplified from *A. baumannii* genomic DNA using primers *blsA*dh and *fur*dh (Table S2), respectively, and subsequently cloned into the BamHI and XhoI sites of Gateway entry vector pENTR3C (Invitrogen) (see Table S1). These CDSs were then transferred using LR Clonase reaction to pGBKT7-Gw and pGADT7-Gw Y2H vectors (Clontech), which were previously adapted to Gateway Technology by the introduction of the Gateway cassette to allow cloning by recombination. Once transformed into yeast cells, these plasmids express fusion proteins to the GAL4 DNA-binding (DB) or activation (AD) domain, under control of the constitutive ADH1 promoter. Proper construction of each plasmid was confirmed by automated DNA sequencing.

#### pWHFUR plasmid construction

A 807-bp fragment harboring *fur* and its predicted promoter was PCR amplified using *A. baumannii* ATCC 19606 genomic DNA and primers PFurF and PFurR (see Table S2), both of which were tailed with BamHI restriction sites. The amplicon was cloned into GEM-T Easy and then subcloned as a BamHI fragment into the cognate site of pWH1266. Proper construction of the complementing pWHFur plasmid was confirmed by automated DNA sequencing.

#### Two-hybrid assays (Y2H)

Y2H analysis were performed essentially as described previously^19^. All the assays were performed on *Saccharomyces cerevisiae* Mav 203 strain (MATa, *leu*2-3,112, *trp*1-901, *his*3-D200, *ade*2-101, *gal*4D, *gal*80D, SPAL10::URA3, GAL1::*lacZ*, HIS3UAS GAL1::HIS3, LYS2, *can*1R and *cyh*2R) transformed with the corresponding expression vectors for Y2H interaction assays. We first tested BlsA and Fur for self-activation in the Y2H system. To do this, the DNA binding domain (DB)-fusion protein expressing vectors (pGBK-X) (X = BlsA or Fur) were transformed into the MaV203 yeast strain. Each MaV203/pGBK-X strain was first transformed with the pGAD-T7 empty vector and these pGBK-X/pGAD containing yeast cells were tested for their ability to activate reporters’ expression without a real X-Y protein–protein interaction (self-activation) and to determine the optimal 3-amino-1,2,4-triazole (3AT) concentration required to titrate the basal *HIS3* reporter expression to be used later in the interaction assays. MaV203/pGBK-X strains were then transformed with each pGAD-Y construction for Y_AD fusion protein expression (again, Y represents BlsA or Fur). Transformations were performed by lithium acetate/single-stranded carrier DNA/polyethylene glycol method^38^ using either one or both Y2H plasmids and plated in the corresponding minimal selective medium (Synthetic Complete medium (SC) without leucine (-leu) for pGAD-Y transformants, SC without tryptophan (-trp) for pGBK-X transformants and SC-leu-trp for both plasmids transformants) and then incubated in the dark at 23 °C for 72 h. Because our previous results suggested that temperature and light may induce conformational changes in the proteins’ structures, we performed all the steps in the dark and at 23 °C, (except when comparing the interaction assays in other conditions). Four to six clones of each pGBK-X/pGAD-Y containing yeasts, four to six self-activation control clones pGBK-X/pGAD and pGBK/pGAD-Y (Y DNA-binding negative control) and two isolated colonies of each of the five yeast control strains (A to E) were patched in the same SC-leu-trp plate and incubated for 48-72 h at 23 °C in the dark to be used as “Master Plate”. To test for the three reporter genes’ expression phenotypes, this Master Plate was then replica plated to SC-leu-trp-his +3AT (3AT was added in the optimal concentration previously determined) and to SC-leu-trp-ura to test for growth in absence of histidine (*his*) and uracil (*ura*), respectively (*his*3 and *ura*3 reporter activation). Each plate was incubated in the corresponding conditions to be tested (dark/light; 23 °C/30 °C) for at least 72 h. For qualitative measurement of β-galactosidase (β-Gal) expression, transformed yeasts were replica plated onto a nitrocellulose filter placed on a YPAD medium plate and incubated in the dark at 23 °C. Once the yeast cells were grown, the nitrocellulose filter was lifted from the agar plate and transferred to a new empty Petri dish. Cells were permeabilized with liquid Nitrogen and then tested for the presence of β-Gal activity by incubation on a filter paper soaked in X-Gal solution (5-bromo-4-chloro-3-indolyl-b-D-galactopyranoside in Z buffer (60 mM Na2HPO4, 40 mM NaH2PO4, 10 mM KCl, 1 mM MgSO4, pH 7.0) under the corresponding conditions to be tested for the interactions (dark/light; 24 °C/30 °C).

#### Analyses of gene expression by qRT-PCR

Retrotranscription and qRT-PCR analysis were done as described in reference^39^, using primers listed in Table S2 in the supplemental information. Data are presented as NRQ (Normalized relative quantities) calculated by the qBASE method^40^, using *recA* and *rpoB* genes as normalizers^6^.

#### Growth of *A. baumannii* under iron deprivation conditions

To test the ability of the *A. baumannii* wild-type and derivative strains used in this work to grow under iron deprivation conditions, 1/100 dilutions of overnight cultures grown in LB Difco or tryptone media were inoculated in the corresponding liquid medium supplemented or not with the indicated iron chelator 2,2′-dipyridyl (DIP) concentrations. Cultures were subsequently grown stagnantly at 23 °C under blue light or in the dark. At the times indicated in the figures, an aliquot was taken to record the A_660_ of the culture.

## Electronic supplementary material

Supplementary information
