# Supplementary material for: BlsA integrates light and temperature signals into iron metabolism through Fur in the human pathogen Acinetobacter baumannii
Source: Sci Rep. 2018 May 16;8:7728. doi: 10.1038/s41598-018-26127-8 (PMC5955987; doi:10.1038/s41598-018-26127-8)
Supplement: Supplementary file 1 — Supplementary information [file 41598_2018_26127_MOESM1_ESM.pdf]

BlsA integrates light and temperature signals into iron metabolism through Fur in the human pathogen *Acinetobacter baumannii*

Marisel R. Tuttobene[1], Pamela Cribb[2], María Alejandra Mussi[1]*.

[1]Centro de Estudios Fotosintéticos y Bioquímicos (CEFOBI- CONICET). 2000. Rosario, Argentina.

[2]Instituto de Biología Molecular y Celular de Rosario (IBR-CONICET). 2000. Rosario. Argentina.

Running title: BlsA interacts with and antagonizes Fur functioning in *Acinetobacter baumannii*.

+Corresponding author: mussi@cefobi-conicet.gov.ar; Phone: 54-341-4371955; Fax: 54-341-4370044

Supplementary information.

Figure S1. Y2H assays of BlsA/BlsA interaction at different conditions: 23ºC light and dark, or 30ºC dark. Each plate contains six clones of MaV203/pGAD-*blsA* transformed with plasmids pGBK-*blsA*, as well as empty plasmid pGBK as negative control. In addition, we included at least six clones of MaV203/pGBK-*blsA* or MaV203/pGBKT*blsA* transformed with empty plasmid pGAD as negative control. Finally, two clones of each interaction-control strain A–E were also included. Pictures show development of blue color (panel A) or yeast growth in Synthetic Complete medium (SC) without leucine, tryptophan and histidine (SC-LWH) supplemented with 25 mM 3-amino-1,2,4-triazole (3AT) (panel B), and SC without leucine, tryptophan and uracil, (SC-LWU) (panel C). For β-galactosidase expression analysis (X-Gal), yeasts were transferred to a nitrocellulose filter, permeabilized and subjected to the X-Gal assay. The scheme on the right side represent the order of yeast streaks on each plate. The assays were performed following procedures described in [16]. Experiments were performed in triplicates and representative results are shown.

Figure S2. Y2H assays of Fur/Fur interaction at different conditions: 23ºC light and dark, or 30ºC dark. Each plate contains six clones of MaV203/pGAD-*fur* transformed with plasmids pGBK-*fur*, as well as plasmid pGBK as negative control. In addition, we included at least six clones of MaV203/pGBKT-*fur* transformed with empty plasmid pGAD as negative control. Finally, two clones of each interaction-control strain A–E were also included. Pictures show development of blue color (first row) or yeast growth in Synthetic Complete medium (SC) without leucine, tryptophan and histidine (SC-LWH) supplemented with 25 mM 3-amino-1,2,4-triazole (3AT) (second row), and SC without leucine,

tryptophan and uracil (SC-LWU) (third row). For β-galactosidase expression analysis (X-Gal), yeasts were transferred to a nitrocellulose filter, permeabilized and subjected to the X-Gal assay. The scheme on the right side represent the order of yeast streaks on each plate. The assays were performed following procedures described in [16]. Panel A experiments were performed in the absence of added iron, while panel B experiments were performed in the presence of external iron addition (0.75 mM $FeCl_3$). Experiments were performed in triplicates and representative results are shown.

Figure S3. Light modulates growth under iron deprived conditions in *A. baumannii* at moderate temperatures. (A) Growth curves of *A. baumannii* ATCC 19606 wild-type and derivative strains used in this study, (B) Growth curves of *A. baumannii* ATCC 17978 wild-type and derivative strains; in tryptone media (tryptone 1%, NaCl 0.5%, agarose 0.3%) supplemented with DIP 150 or 175 µM and incubated stagnantly at 23°C under blue light or in the dark.

Figure S4. *blsA* is expressed in a temperature-dependent manner in *Acinetobacter baumannii*. Estimation by RT-qPCR of the expression levels of *blsA* in ATCC 19606 wild-type at 23ºC and 30ºC under blue light (L) or in the dark (D). The data shown are mean ± SD of normalized relative quantities (NRQ) obtained from transcript levels of *blsA*, in samples grown in LB at the indicated DIP concentrations under blue light or in the dark at 23ºC or 30ºC, measured in at least three biological replicates. Different letters indicate significant differences as determined by ANOVA followed by Tukey’s multiple comparison test ($p<0.05$).

Table S1. Bacterial and yeast strains used in this work.

Strain/plasmid	Relevant characteristic	Source or reference
A. baumannii		
ATCC 19606^T	Clinical isolate, type strain	ATCC
ATCC 17978	Clinical isolate	ATCC
ATCC 17978 Δ*blsA*	*blsA*::aph derivative of 17978; Km^r	[4]
ATCC 17978 Δ*blsA* pWHBlsA	17978 Δ*blsA* harboring plasmid pWHBlsA; Km^r Amp^r	[4]
ATCC 17978 Δ*blsA* pWH1266	17978.OR harboring pWH1266; Km^r Tet^r Amp^r	[4]
ATCC 19606 pWH1266	19606 harboring pWH1266; Tet^r Amp^r	This work
ATCC 19606 pWHFur	19606 harboring pWHFur, Amp^r	This work
ATCC 19606 Δ*blsA*	*blsA*::aph derivative of 19606; Km^r	[9]
ATCC 19606 Δ*blsA* pWHBlsA19	*blsA*::aph derivative of 19606 harboring pWHBlsA19; Km^r Amp^r	[6]
ATCC 19606 Δ*blsA*	*blsA*::aph derivative of	[6]

pWH1266	19606 harboring pWH1266; Km^r Tet^r Amp^r	
ATCC 19606 Δ*blsA* pWHFur	*blsA*::aph derivative of 19606 harboring pWHFur, Km^r Amp^r	This work
E. coli		
DH5α	Used for DNA recombinant methods	Gibco-BRL
BL21 (DE)	Overexpression of His-tagged BlsA	Novagen
Saccharomyces cerevisiae		
Mav 203 strain	MATa, *leu*2-3,112, *trp*1-901, *his*3-D200, *ade*2-101, *gal*4D, *gal*80D, SPAL10::URA3, GAL1::*lacZ*, HIS3UAS GAL1::HIS3, LYS2, *can*1R and *cyh*2R	Thermofisher
**Plasmids**		
pBluescript	PCR cloning vector; Ampr	Promega
pEBlsA	pET-TEV harboring a wild type copy of *blsA*; Kmr	[4]

pWH1266	*E. coli-A. baumannii* shuttle vector; Ampr Tcr	[40]
pWHBlsA	pWH1266 harboring wild-type copy of *blsA* from ATCC 17978 expressed under its own promoter; Ampr	[4]
pWHBlsA19	pWH1266 harboring wild-type copy of *blsA* from ATCC 19606 expressed under its own promoter; Ampr	(6)
pWHFur	pWH1266 harboring wild-type copy of *fur* from ATCC 19606 expressed under its own promoter; Ampr	This work
pENTR3C	Gateway system entry-vector	Invitrogen- Thermofisher
PGAD-T7-GW	Y2H AD-fusion vector, adapted to Gateway System	Clontech, [16]
PGBK-T7-GW	Y2H DB-fusion vector, adapted to Gateway System	Clontech, [16]

Table S2. Primers used in this study.

**Name/No.**	**Nucleotide sequence**	**Source or reference**
RecAF.rt	5´- TACAGAAAGCTGGTGCATGG-3´	[4]
RecAR.rt	5´- TGCACCATTTGTGCCTGTAG -3´	[4]
RpoBF.rt	5'- CAGAAGTCACGCGAAGTTGAAGGT-3'	[6]
RpoBR.rt	5'- AACAGCACGCTCAACACGAACT-3'	[6]
*bauA*F.rt	5'- AAATGTTTGGCCGCGTTGAGGT- 3'	This work
*bauA*R.rt	5'- CAATCGTGCAAACGGTTCATCAGC- 3'	This work
*basD*F.rt	5'- TGCACAGATTGCTCCCGTGGTATT- 3'	This work
*basD*R.rt	5'- ACTTGCGGCCCTTGTGAAATGA - 3'	This work
PFurF	5'-GGATCC CATGCCGACCTGTACTTGAG- 3'	This work
PFurR	5'- GGATCC GGTCCTGATCAATTATCAGACG- 3'	This work
*bauD*F.rt	5'- TTACAGCCAGCCGTATTCCTCGTT- 3'	This work
*bauD*R.rt	5'- CCCGACGTTTCCGGTTCAACAAAT- 3'	This work
*basE*F.rt	5´- TGACCAACCTTTGACGCGCATT -3'	This work
*basE*R.rt	5' - AAGCAGTATCACCCTTGCCCAAAC -3'	This work
*basA*F.rt	5' - TACAGCTTTGGCATGTGCGCTT -3'	This work
*basA*R.rt	5' - TCACCACAACATCTGCCCGACTAT -3'	This work
FurF.rt	5'- GCTGGACTTAAAGTTACCCTTCCACG-3'	This work
FurR.rt	5'- ACACACGGTAAACTGTCGCAAGTC-3'	This work
*blsA*dhF	5'- GGATCCATGAACGTTCGCCTGTGT-3'	This work

*blsA*dhR	5'- CTCGAGTGCTAGAACGGGTTTACTC-3'	This work
*fur*dhF	5'- GGATCCATGCCTATTTCCAATCAAG-3'	This work
*fur*dhR	5'- CTCGAGACTTATTTCTTGCGCAATG-3'	This work

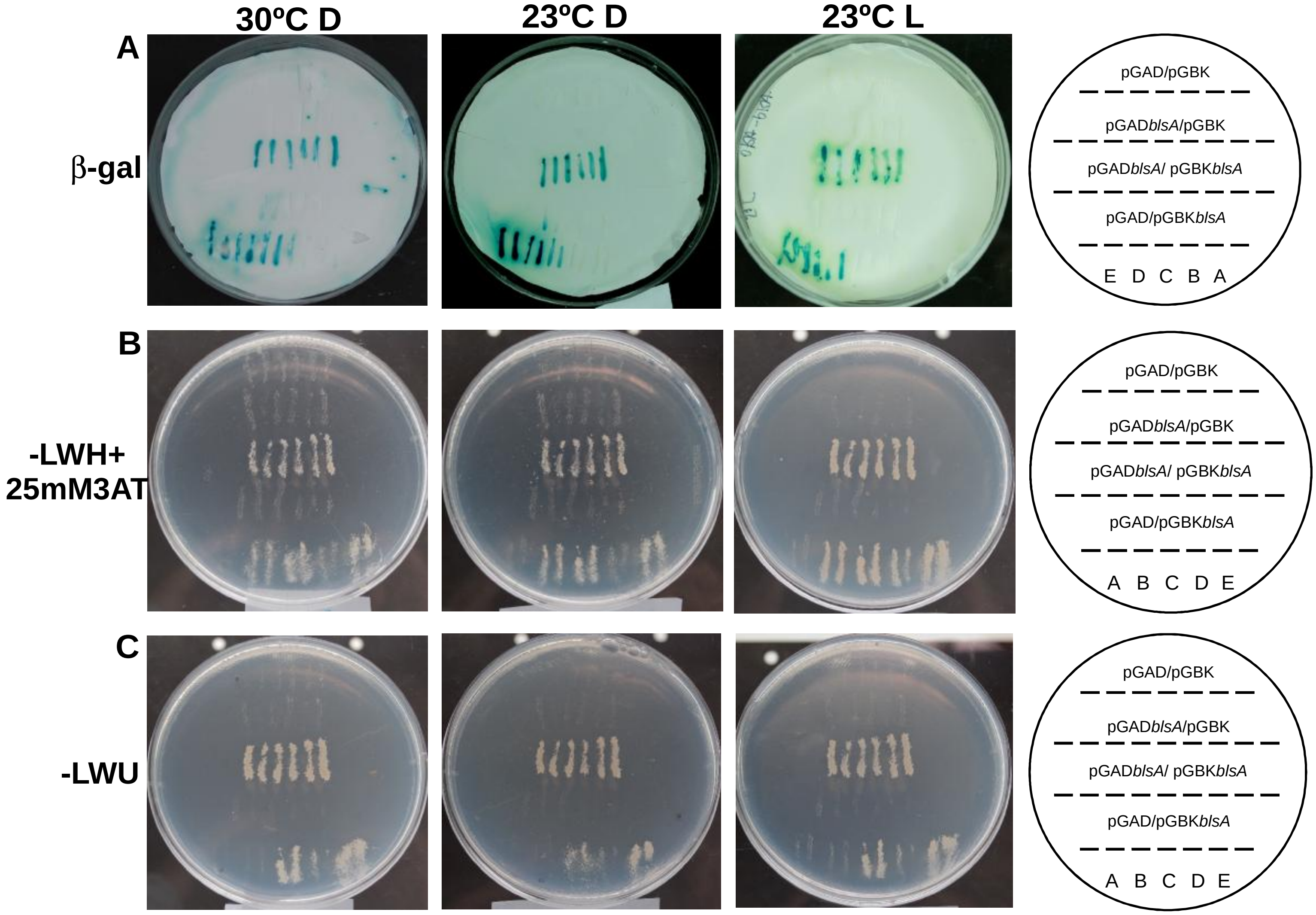
30ºC D
23ºC D
23ºC L
A
β-gal
B
-LWH+
25mM3AT
C
-LWU
pGAD/pGBK
pGADblsA/pGBK
pGADblsA/ pGBKblsA
pGAD/pGBKblsA
E D C B A
A B C D E

Figure S1

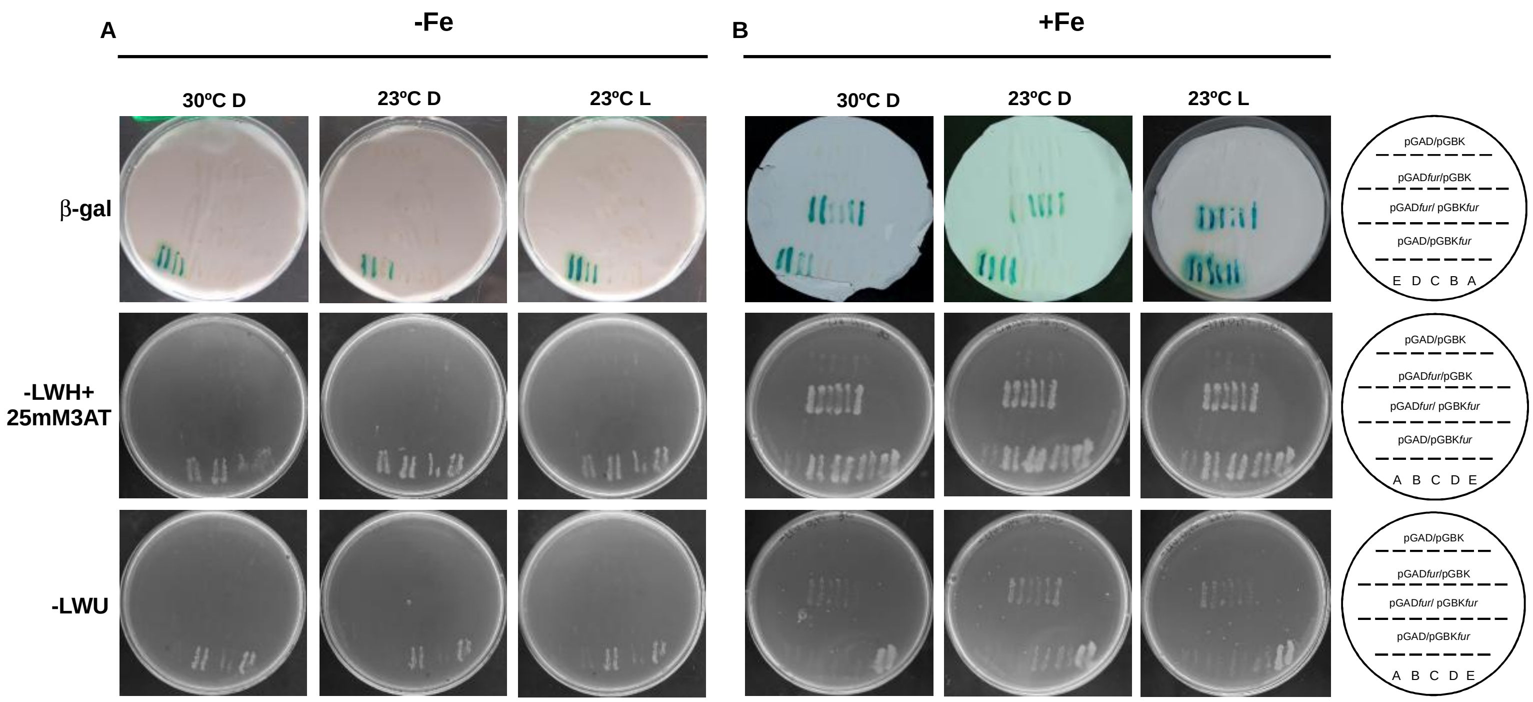
A
-Fe
B
+Fe
30ºC D
23ºC D
23ºC L
30ºC D
23ºC D
23ºC L
β-gal
-LWH+
25mM3AT
-LWU
pGAD/pGBK
pGADfur/pGBK
pGADfur/ pGBKfur
pGAD/pGBKfur
E D C B A
A B C D E

Figure S2

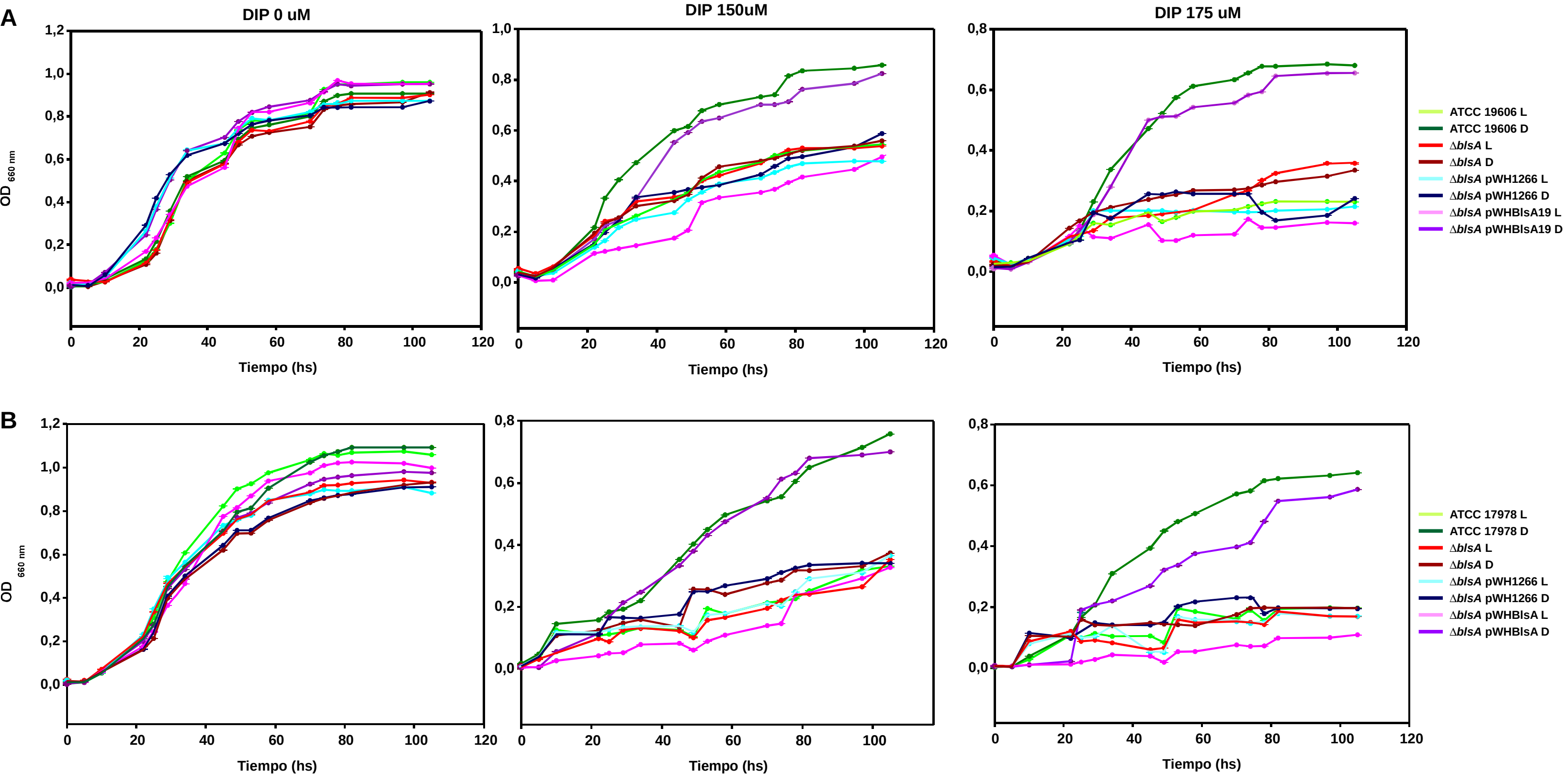
A
DIP 0 uM
DIP 150uM
DIP 175 uM
OD 660 nm
Tiempo (hs)
ATCC 19606 L
ATCC 19606 D
ΔblsA L
ΔblsA D
ΔblsA pWH1266 L
ΔblsA pWH1266 D
ΔblsA pWHBlsA19 L
ΔblsA pWHBlsA19 D
B
ATCC 17978 L
ATCC 17978 D
ΔblsA L
ΔblsA D
ΔblsA pWH1266 L
ΔblsA pWH1266 D
ΔblsA pWHBlsA L
ΔblsA pWHBlsA D

Figure S3

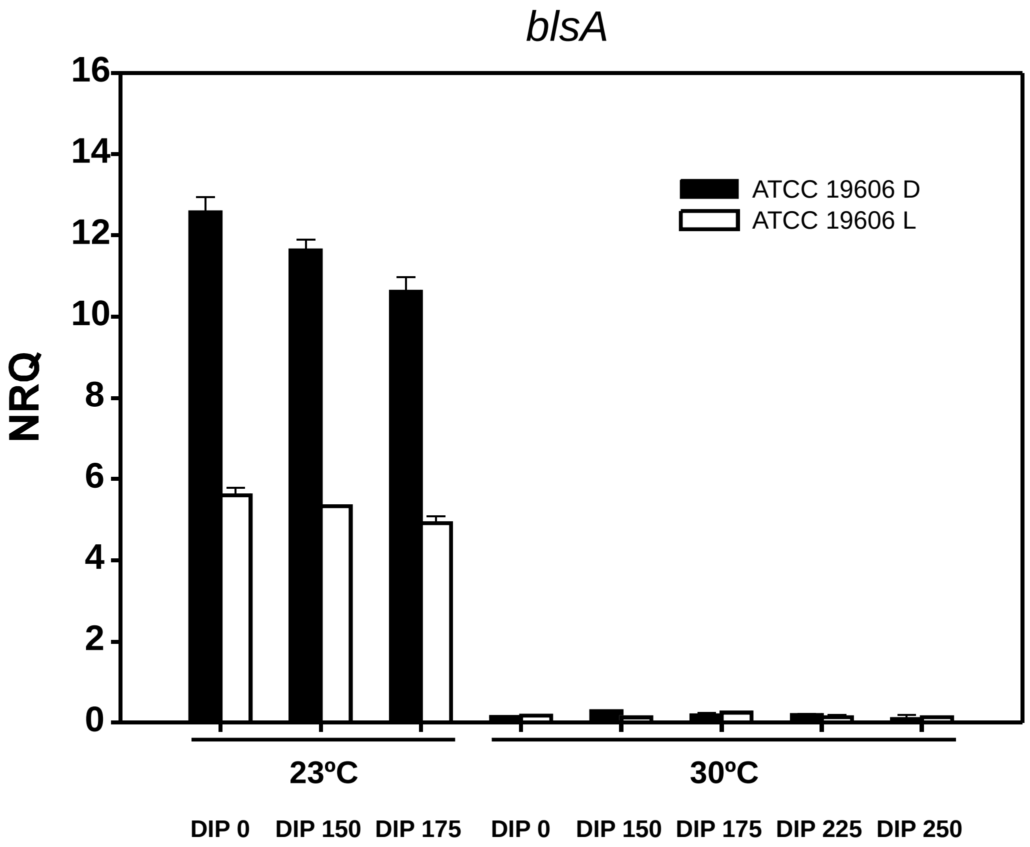
blsA
NRQ
16
14
12
10
8
6
4
2
0
ATCC 19606 D
ATCC 19606 L
23ºC
30ºC
DIP 0
DIP 150
DIP 175
DIP 0
DIP 150
DIP 175
DIP 225
DIP 250

Figure S4
